# Reduction in focal ictal activity following transplantation of MGE interneurons requires expression of the GABA_A_ receptor α4 subunit

**DOI:** 10.3389/fncel.2015.00127

**Published:** 2015-04-09

**Authors:** Manoj K. Jaiswal, Sotirios Keros, Mingrui Zhao, Melis Inan, Theodore H. Schwartz, Stewart A. Anderson, Gregg E. Homanics, Peter A. Goldstein

**Affiliations:** ^1^C.V. Starr Laboratory for Molecular Neuropharmacology, Department of Anesthesiology, Weill Cornell Medical CollegeNew York, NY, USA; ^2^Department of Pediatrics, Weill Cornell Medical CollegeNew York, NY, USA; ^3^Department of Neurological Surgery, Weill Cornell Medical CollegeNew York, NY, USA; ^4^Brain and Mind Research Institute, Weill Cornell Medical CollegeNew York, NY, USA; ^5^Department of Psychiatry, Children’s Hospital of Philadelphia, University of Pennsylvania School of MedicinePhiladelphia, PA, USA; ^6^Department of Anesthesiology, University of PittsburghPittsburgh, PA, USA

**Keywords:** GABA_A_ receptor, extrasynaptic, α4 subunit, interneuron, epilepsy, cortex

## Abstract

Despite numerous advances, treatment-resistant seizures remain an important problem. Loss of neuronal inhibition is present in a variety of epilepsy models and is suggested as a mechanism for increased excitability, leading to the proposal that grafting inhibitory interneurons into seizure foci might relieve refractory seizures. Indeed, transplanted medial ganglionic eminence interneuron progenitors (MGE-IPs) mature into GABAergic interneurons that increase GABA release onto cortical pyramidal neurons, and this inhibition is associated with reduced seizure activity. An obvious conclusion is that inhibitory coupling between the new interneurons and pyramidal cells underlies this effect. We hypothesized that the primary mechanism for the seizure-limiting effects following MGE-IP transplantation is the tonic conductance that results from activation of extrasynaptic GABA_A_ receptors (GABA_A_-Rs) expressed on cortical pyramidal cells. Using *in vitro* and *in vivo* recording techniques, we demonstrate that GABA_A_-R α4 subunit deletion abolishes tonic currents (I_tonic_) in cortical pyramidal cells and leads to a failure of MGE-IP transplantation to attenuate cortical seizure propagation. These observations should influence how the field proceeds with respect to the further development of therapeutic neuronal transplants (and possibly pharmacological treatments).

## Introduction

The treatment of seizures by transplantation of inhibitory interneurons into epileptic foci is an area of considerable interest (Anderson and Baraban, 2012). Various transplantation strategies have been employed to suppress epileptic activity (Raedt et al., 2007), including the use of interneuron precursors harvested from the embryonic MGE (Alvarez-Dolado et al., 2006; Baraban et al., 2009; Waldau et al., 2010; De la Cruz et al., 2011; Henderson et al., 2014). Transplanted MGE-IPs differentiate into mature GABAergic interneurons that provide inhibitory synaptic and extrasynaptic GABAergic transmission onto cortical pyramidal neurons (Sultan et al., 2013). This inhibition is associated with a reduction in seizure activity (Alvarez-Dolado et al., 2006; Baraban et al., 2009).

Extrasynaptic activity, a response based on a distinct population of GABA_A_ receptors (GABA_A_-Rs), is of great significance in mediating neuronal inhibition (Lee and Maguire, 2014). Immunohistochemical (Pirker et al., 2000; Chandra et al., 2006; Hörtnagl et al., 2013) and pharmacologic (Brown et al., 2002; Krook-Magnuson and Huntsman, 2005; Drasbek and Jensen, 2006; Drasbek et al., 2007; Vardya et al., 2008) data indicate that the GABA_A_-R α4 subunit contributes to the formation of extrasynaptic GABA_A_-Rs in Layer 2/3 and Layer 4 cortical neurons in the rodent brain. In deeper neurons (Layer 5), the α5 subunit contributes to the tonic current in both neonatal (Sebe et al., 2010) and juvenile (Yamada et al., 2007) animals. Importantly, a tonic current has also been observed in Layer V-VI pyramidal cells from human neocortex, where less than a third of the tonic current results from activation of GABA_A_-Rs containing the α5 subunit (Scimemi et al., 2006), with the subunit-basis of the remainder of the current unknown. It is possible, however, that the non-α5 subunit-mediated current is generated by GABA_A_-Rs containing the α4 subunit as α4 subunit mRNA is also present in those cells (Petri et al., 2003).

GABA-mediated tonic inhibition appears to inhibit seizure activity as the anticonvulsants vigabratin and tiagabine (which inhibit GABA-transamninase and GABA reuptake, respectively) increases the concentration of GABA in the brain (Petroff et al., 1996; Richards and Bowery, 1996); importantly, this increase results, in part, from non-vesicular release of GABA (Wu et al., 2003). Deletion of the GABA_A_-R δ subunit, which co-assembles with the α4 subunit (Sur et al., 1999; Jia et al., 2005) and contributes to the formation of extrasynaptic GABA_A_-Rs (Nusser et al., 1998; Sassoè-Pognetto et al., 2000; Brickley et al., 2001; Nusser and Mody, 2002; Jia et al., 2005), is associated with a decrease in α4 expression (Peng et al., 2002) and has pro-convulsant effects (Spigelman et al., 2002). In dentate gyrus cells in a rat TLE model, there was a significant decrease in the neurosteroid-sensitive tonic current which occurred in combination with a decrease in the surface expression of the δ subunit (Peng et al., 2004; Rajasekaran et al., 2010), suggesting a close relationship between extrasynaptic GABA_A_-Rs and seizure activity. In a parallel fashion, there is a shift in α4 expression from the extrasynaptic to synaptic membrane in the rat TLE model (Sun et al., 2007), consistent with a loss of extrasynaptic GABA_A_-Rs. In a rat traumatic brain injury model, there is a significant decrease in the tonic current in semilunar granule cells (SGCs; which are excitatory neurons located in the dentate inner molecular layer), and the loss of the tonic current is associated with a significant increase in input resistance and a significant increase in SGC excitability (Gupta et al., 2012). In the epileptic mutant mouse stargazer, there is a preferential loss of extrasynaptic GABA_A_-Rs (Payne et al., 2006, 2007). More compellingly, mutations associated with both febrile seizures and childhood absence epilepsy compromise surface expression of GABA_A_-Rs and reduce tonic GABA currents but do not affect GABA-mediated synaptic events (Eugène et al., 2007). Finally, at least one clinically relevant AED, gabapentin, has been shown to enhance the tonic current in hippocampal neurons (Cheng et al., 2006). Thus, activation of extrasynaptic GABA_A_-Rs represents a novel approach to treating seizures arising in cortical, rather than subcortical, structures (Cope et al., 2009).

Multiple animal models exist for studying seizure activity *in vivo* (Pitkänen et al., 2006), including application of the transient potassium current blocker, 4-aminopyridine (4-AP; Barna et al., 2000; Bahar et al., 2006; Ma et al., 2009; Zhao et al., 2009, 2015; Medina-Ceja and Ventura-Mejía, 2010; Salazar and Tapia, 2012; Yu et al., 2014). We have chosen an acute, rather than chronic, focal seizure mouse model for several reasons. First, a model of partial onset epilepsy is required since MGE-IP transplants are intended as a focal therapy. Second, the ictal events induced with 4-AP last one to two minutes and appear electrographically exactly like partial onset human epilepsy (Avoli, 1990; Perreault and Avoli, 1991; Heinemann et al., 2006), beginning with low-voltage, fast-activity and progressing to paroxysmal spike-and-wave activity (Schwartz and Bonhoeffer, 2001). Third, ictogenesis is reliable and easy to induce. Together, these attributes make the 4-AP acute seizure model a viable and useful paradigm for examining both underlying mechanism(s) as well as potential treatment efficacy (Löscher, 2011).

We hypothesized that the observed seizure-limiting effect of MGE-IP transplantation results from activation of extrasynaptic GABA_A_-Rs expressed on cortical pyramidal cells. Determining whether the reduction in ictal activity following MGE-IP transplantation arises from synaptic or extrasynaptic activity will guide the development of transplant-based therapies and potential pharmacological treatments for medically refractory seizure disorders.

## Methods

All animal experiments were performed in accordance with Institutional and Federal guidelines. The principles in the ARRIVE Guidelines from The National Centre for the Replacement, Refinement and Reduction of Animals in Research (London, UK)^1^ were considered when planning the experiments. To avoid periodic alterations in specific GABA_A_R subunits during the estrous cycle in mice, which are associated with cyclic changes of tonic inhibition and seizure susceptibility (Maguire et al., 2005), only male mice were used for *in vitro* and *in vivo* experiments.

### *In Vivo* Transplantation of MGE Cells

Pan-green fluorescent protein (GFP)-expressing transgenic mice were maintained on a CD1 background. At 13 days of gestation (E13.5), dams were sacrificed, GFP+ embryos harvested, and their brains removed. MGE-IP cells were obtained from 250 µm sections by mechanical dissociation after separating the dorsal and ventral MGE from adjacent brain regions (Figure 2A). Cells were re-suspended in ice-cold neurobasal/B27 medium until transplantation (De la Cruz et al., 2011).

Post-natal GABA_A_-R α4 subunit deletion (α4^−/−^) mice or wild-type (α4^+/+^) male littermates at P30–40 were anesthetized with sevoflurane and mounted in a stereotaxic frame. Using a nanoinjector, 1.5 × 10^4^ MGE-IP cells in 1.5–2.0 µl were injected into the motor cortex (coordinates relative to Bregma: anteroposterior (*AP*) −0.7 mm, mediolateral (*ML*) −1.0 mm, and dorsoventral (*DV*) −1.0 mm) through a small burr hole. As a sham control, in a separate group of animals, an equivalent volume and number of MGE cells killed by freezing and thawing was injected in the same location.

### *In Vitro* Electrophysiology and Analysis

Whole-cell patch clamp recordings were made from visually identified cortical layer 2/3 neurons in acutely prepared 300-µm thick coronal brain slices (using IR/DIC microscopy) from GABA_A_-R α4^−/−^ mice and α4^+/+^ male littermates using a Nikon Eclipse FN1 microscope equipped with a 4× objective and a 40× water immersion objective and a high-sensitivity monochrome digital camera (DS-Qi1, Nikon). Pyramidal cells were selected based on pyramidal shape, apical dendrite, and distance (200–300 µm) from pia. Standard whole-cell patch clamp recordings were made in either the voltage or current clamp configuration using a Multiclamp 700 amplifier (Molecular Devices, Sunnyvale, CA) and pClamp 10.2 software (Molecular Devices). Data were acquired at 10 kHz, filtered at 2.2 kHz, and digitized using a Digidata 1440A A/D interface (Molecular Devices). All voltage clamp experiment recordings were performed at −70 mV; neurons having resting membrane potential greater than −45 mV were omitted from the study. Liquid junction potential (+3.8 mV) was not corrected. Series resistance was less than 25 MΩ following membrane rupture and with whole-cell configuration established; data were excluded if resistance changed by more than 20% over the course of the recording. For analysis of sIPSC decay times, currents were fit with a monoexponential function and the 80–20% decay time recorded.

#### Drugs and Solutions

Salts and D-(-)-2-amino-5-phosphonopentanoic acid (AP5), (±)-3-piperidine carboxylic acid (nipecotic acid; NPA) were from Sigma-Aldrich (St. Louis, MO); bicuculline methochloride, 6-cyano-7-nitroquinoxaline-2, 3-dione (CNQX), 11, 12, 13, 13a-tetrahydro-7-methoxy-9-oxo-9*H*-imidazo [1, 5-*a*] pyrrolo [2, 1-*c*] [1, 4] benzodiazepine-1-carboxylic acid, ethyl ester (L655, 708), and (2*S*)-(+)-5, 5-dimethyl-2-morpholineacetic acid (SCH 50911) were from Tocris (Bristol, UK). CNQX, AP5, bicuculline, and NPA were dissolved in distilled water to a stock concentration of 10, 20, 25 and 100 mM, respectively. L655, 708 and SCH 50911 were dissolved in dimethyl sulfoxide (DMSO) to a stock concentration of 5 and 10 mM, respectively. Stock solutions were kept at −20°C and added to the extracellular recording solution prior to recording.

The various solutions contained (in mM):

Slicing solution: 250 sucrose, 2 KCl, 5 MgCl_2_, 0.5 CaCl_2_, 1.25 NaH_2_PO_4_, 26 NaHCO_3_ and 10 D-glucose (ice-cold) saturated with 95% O_2_/5% CO_2_.

Incubation extracellular solution: 126 NaCl, 3.6 KCl, 5 MgCl_2_, 0.5 CaCl_2_, 1.2 NaH_2_PO_4_, 26 NaHCO_3_ and 10 D-glucose; pH 7.4 when saturated with 95% O_2_/5% CO_2._ Incubation solution was initially at 36°C, and after 40 min the temperature of the incubation solution was gradually lowered and slices were maintained at room temperature (22–24°C); all subsequent electrophysiology experiments were performed at room temperature.

Extracellular recording solution: 126 NaCl, 3.6 KCl, 1.5 MgCl_2_, 2.5 CaCl_2_, 1.2 NaH_2_PO_4_, 26 NaHCO_3_, 10 D-glucose saturated with 95% O_2_/5% CO_2_. The extracellular recording solution also contained: AP5 (20 µM), CNQX (10 µM), and SCH 50911 (10 µM). When present, the final concentrations of NPA, bicuculline, and L655, 708 were, respectively: 1 mM (a concentration which completely blocks GABA reuptake (Keros and Hablitz, 2005)), 50 µM, and 250 nM.

Intracellular solution: 135 KCl, 10 HEPES, 2 Mg-ATP, 0.2 Na-GTP, 0.5 EGTA; pH 7.4.

The magnitude of the tonic GABA-mediated current was calculated by measuring the difference in the baseline whole-cell current in the absence and presence of bicuculline (50 µM; a concentration which is sufficient to block all GABA_A_-R-mediated currents in brain slices (Yamada et al., 2007)). The current clamp configuration was used to measure membrane potentials. Tonic current amplitude was analyzed as a function of genotype, and results were compared using either unpaired Student’s *t*-test or the Mann-Whitney Rank Sum Test (if the data did not pass either normality or equal variance tests) as appropriate; *p* < 0.05 was considered significant. Data are presented as mean ± SEM.

### *In Vivo* Electrophysiology

Methods employed here are similar to those previously reported (De la Cruz et al., 2011). Briefly, thirty to forty days post-transplantation, adult male mice were anesthetized with isoflurane and mounted in a stereotaxic frame. Electrodes were inserted into somatosensory cortex through a small cranial window after opening the dura. A custom-designed chamber was affixed to the skull and filled with silicone oil. Ictal discharges were induced by injecting 4-AP (15 mM in 0.9% saline, 0.5 µl) through a single-barreled glass microelectrode placed 2 mm away from the site of the transplant. The local field potential (LFP) was recorded with two electrodes (see Figure 2E for general configuration). Ictal onset was recorded from the same electrode used for 4-AP injection (LFP1). Ictal propagation was recorded from the site of transplantation (LFP2), which was identified by the injection burr hole. A reference electrode (secured by one miniature stainless steel screw) was placed into the skull above the cerebellum. LFPs were amplified, filtered (0.1–1,000 Hz) and digitized at 1 kHz using two AC/DC differential amplifiers (Model 3000, A-M systems, Carlsborg, WA). Data were recorded on a PC using a CED Power 1401 and Spike2 software (Cambridge Electronic Design, Cambridge UK). The amplitude and duration of ictal activity was recorded at each of the two LFP electrodes, one in the 4-AP focus (LFP1) and the other in the region of the cell transplants (LFP2) to determine propagation efficacy.

### 4-AP *in Vivo* Data Analysis

Off-line analysis was performed using custom analysis software written in Matlab (MathWorks, Natick, MA) (De la Cruz et al., 2011). Seizure onset was determined by visual analysis of the LFP1 recording based on the morphology of the seizures. In the 4-AP seizure model, most seizures start with a large spike-and-wave, followed by a recruiting rhythm that evolves into repetitive spike-and-wave discharges that dissipate with progressive decrease in amplitude and increase in inter-spike interval (Zhao et al., 2009). Consistent with our prior experience, such activity persisted for up to 2 h (data not shown). This large initial spike was used as the marker of ictal onset; termination of ictal activity (offset) was determined by the cessation of the spike-and-wave discharges and the return to baseline, pre-ictal activity. The time windows between ictal onset and offset was used for the further analysis. The average seizure duration at the 4-AP injection electrode (LFP1 recording site) was 33.6 ± 10.8 s (315 seizures, 20 mice). After setting a threshold 2 × SD above baseline activity, total LFP (ΣLFP) power was calculated as the integral of the LFP power during each period of ictal activity (as demarcated by ictal onset and offset as defined above). Baseline activity was measured over a 2 s epoch prior to the initial “onset” spike.

The ratios of ΣLFP power and duration from LFP1 were compared to LFP2 to establish the effect of MGE cells transplantation on each epileptic event. The power and duration calculations were performed using custom-made analysis program based on Matlab. The averaged ratio result of all ictal discharges from an individual mouse was used for the further group analysis. For fast Fourier transform (FFT) analysis between LFP1 and LFP2, cross power spectrum and coherence calculations were performed using “cpsd” and “mscohere” functions in Matlab (Bronzino, 1984; Davey et al., 2000). Each computation used Welch’s averaged periodogram method (Welch, 1967) with NFFT of 256 and a Hanning window of the same size. Comparisons of the effects of transplantation on the power ratio between the two groups, α4^+/+^ and α4^−/−^, as a percentage of the power ratio for control killed cell injections were performed with two-way ANOVA with Tukey-Kramer *post hoc* analysis for multiple comparisons (SPSS Statistics, IBM). Repeated-measures ANOVA was used for analysis of both the cross spectral density estimates and magnitude-squared coherence (MSC).

4-AP injected at site 1 (LFP1) simultaneously induces ictal activity at *both* LFP1 and LFP2 recording sites under control conditions (Figures 3A,C); this is in contrast to the situation following MGE-IP transplantation in wild-type mice, where ictal activity is significantly reduced at LFP2, but is preserved at the LFP1 site of 4-AP injection (Figure 3B). Thus, ictal activity induced by 4-AP injection reflects ictal propagation by cortical spread rather than 4-AP diffusion.

### Immunohistochemistry

Mice transplanted with MGE-IPs were intracardially perfused with phosphate buffered saline (PBS) followed by 4% paraformaldehyde prepared in PBS immediately after 4-AP induced seizure experiments. Brains were stored in 4% paraformaldehyde for at least 48 h to allow for proper fixation, after which 50 µm-thick coronal sections were prepared. Free floating sections were placed in blocking solution (5% bovine serum albumin (BSA) in PBS with 3% triton) for 1 h followed by incubation in primary antibody (chicken anti-GFP; 1:1000; Abcam, Cambridge, MA) in blocking solution for 72 h at 4°C. Sections were then incubated in secondary antibody (Alexa-488 goat anti-chicken; 1:250; Life Technologies, Grand Island, NY) for 2 h at room temperature, washed, mounted on a glass slide with aqueous mounting medium (Fluoromount; Southern Biotech, Birmingham, AL) and visualized using epifluorescent microscopy.

## Results

**Figure 1:**
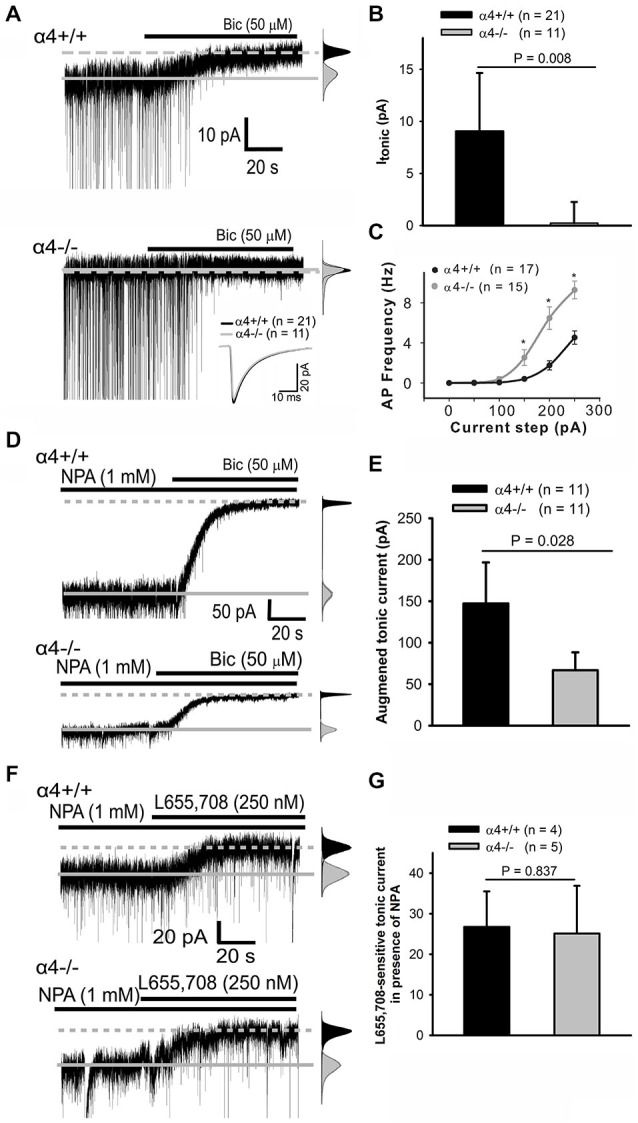
**α_4_ subunit-containing GABA_A_-Rs are the basis of I_tonic_ in cortical pyramidal neurons. (A)** Whole-cell currents from α4^+/+^ (**top trace**) and α4^−/−^ (**bottom trace**) layer II/III pyramidal cells in the absence and presence of bicuculline (bic); traces truncated here and in **d top** for clarity. sIPSCs are readily visible; inset shows overlay of averaged ensemble sIPSCs. The solid line indicates average baseline current without bicuculline while the dashed line indicates average baseline current in the presence of bicuculline. All-points histograms (gray: control; black, bicuculline) highlight the shift in the baseline current in the α4^+/+^, but not α4^−/−^, neuron. **(A, right) (B)** Bar graph summarizing I_tonic_ data. **(C)** Input-output curves for evoked spike firing in neurons from α4^+/+^ and α4^−/−^ mice. * *p* < 0.05 (Mann-Whitney Rank Sum test).** (D, left)** Representative traces demonstrating that nipecotic acid (NPA) enhances the persistent current in both α4^+/+^ (**top trace**) and α4^−/−^ (**bottom trace**) neurons. All-points histograms here (and in **F**) were constructed as in Panel **(A). (E)** The effects of NPA are significantly different between genotypes (Mann-Whitney Rank Sum Test). **(F, left)** Representative traces demonstrating that superfusion of L655, 708 in the presence of NPA has a comparable effect on persistent currents in α4^+/+^ (**top trace**) and α4^−/−^ neurons (**bottom trace**); this effect is not significant between genotypes (unpaired *t*-test) **(G)**.

GABA activates synaptic GABA_A_-Rs, which underlie “phasic” inhibitory postsynaptic currents (IPSCs), as well as extrasynaptic GABA_A_-Rs, which mediate persistent “tonic” currents (Lee and Maguire, 2014). A limited repertoire of GABA_A_-Rs generate I_tonic_, including those containing the α4 subunit (Lee and Maguire, 2014). Evidence suggests that the GABA_A_-R α4 subunit contributes to I_tonic_ in cortical neurons (Chandra et al., 2006; Drasbek et al., 2007). Electrophysiological confirmation of this hypothesis is provided in Figure 1; the average tonic current in α4^+/+^ pyramidal neurons was 9.0 ± 2.8 pA (*n* = 21). Deletion of the α4 subunit abolishes I_tonic_ (Figures 1A,B); the average tonic current in α4^−/−^ pyramidal neurons was 0.2 ± 1.0 pA (*n* = 11). Spontaneous IPSCs (sIPSCs) were unaffected [average (from >2,000 events/genotype) amplitude, decay time, and frequency for α4^+/+^ and α4^−/−^ neurons are, respectively: −48.2 ± 3.2 and −44.4 ± 2.6 pA, 11.5 ± 0.6 and 10.2 ± 0.5 ms, and 4.2 ± 0.3 and 4.4 ± 0.3 Hz; Figure 1A inset]. Deletion of the α4 subunit results in neuronal hyper-excitability (Figure 1C), consistent with a lower threshold for chemically-induced seizures (Chandra et al., 2008). Interestingly, however, the input resistance (R_in_) was not significantly different between the two genotypes (α4^+/+^, 125.8 *±* 11.9 MΩ, *n* = 20 and α4^−/−^, 118.1 *±* 6.1 MΩ, *n* = 20; *p* = 0.735, Mann-Whitney Rank Sum test as data did not pass normality test). Preservation of R_in_ despite the loss of the tonic GABA_A_-R α4 subunit-dependent Cl^−^ conductance could result from compensatory changes in a voltage-independent K^+^ conductance as is seen in response to loss of the tonic current generated by GABA_A_-Rs containing the α6 subunit (Brickley et al., 2001).

The GABA_A_-R α5 (but not α1) subunit has been proposed to contribute to I_tonic_ in cortical neurons (Drasbek et al., 2007; Sebe et al., 2010; Lee and Maguire, 2014). This was tested pharmacologically by increasing extracellular GABA concentrations with nipecotic acid (NPA) in order to maximize the detection of all possible GABA_A_-Rs to I_tonic_. Inhibition of GABA reuptake with NPA significantly (Mann-Whitney Rank Sum test) enhanced persistent currents in layer 2/3 pyramidal cells from α4^+/+^ (147.3 ± 24.8 pA, *n* = 11) as compared to α4^−/−^ mice (66.7 ± 10.8 pA, *n* = 11; Figures 1D,E). The NPA-induced “tonic” current in α4^−/−^ neurons could result from either α5 up-regulation and/or persistent activation of low affinity synaptic GABA_A_-Rs. To evaluate the role of the α5 subunit in generating this current, the GABA_A_-R α5 subunit-selective inverse agonist L655, 709 was co-applied with NPA and found to have the same effect on I_tonic_ in both α4^+/+^ (26.8 ± 4.4 pA, *n* = 4) and α4^−/−^ (25.1 ± 5.9 pA, *n* = 5) mice (Figures 1F,G), indicating that there is no compensatory up-regulation of the α5 subunit in α4^−/−^ mice. The NPA-induced “tonic” current observed in α4^−/−^ neurons after α5 subunit blockade likely reflects persistent activation of incompletely desensitized synaptic receptors (Keros and Hablitz, 2005).

**Figure 2:**
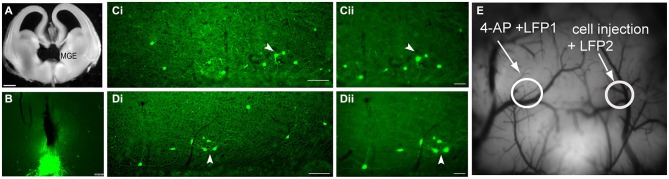
**Transplantation of green fluorescent protein (GFP)-positive embryonic medial ganglionic eminence (MGE) cells into the adult cortex results in long-term survival, migration, and arborization. (A)** MGE dissection in an E13.5 embryo. **(B)** Photomicrograph of a coronal brain section from an α4^+/+^ mouse demonstrating residual clustering of MGE neurons 30 days post-transplantation at the site of injection (see Methods for stereotaxic coordinates). Scale bar: 100 µm. **(C,D)** Low-power photomicrographs (scale bar: 100 µm) of coronal brain sections from α4^+/+^ **(Ci)** and α4^−/−^ **(Di)** mice demonstrating that transplanted GFP^+^ neurons migrate from the site of injection and form comparable arborization patterns. The transplanted MGE progenitors migrated throughout the cortex, and as far as 1 mm from the injection site. **(Cii–Dii)** Higher magnification images (scale bar: 50 µm) of the area indicated by the white arrowhead in **(Ci,Di)** shows that the transplanted interneurons have processes similar to mature neurons. **(E)** Image of the cortical surface indicating the locations of the LFP1 electrode, the 4-AP injection site, the LFP2 electrode, and the MGE/control cell injection site.

We have previously demonstrated that transplantation of medial ganglionic eminence interneuron precursors (MGE-IPs) harvested from embryonic (E13.5) brain (Figure 2A) and transplanted into the adult cortex results in long-term survival, migration, and differentiation into interneurons (De la Cruz et al., 2011). A population of transplanted MGE-IPs remains at the site of injection (Figure 2B), and consistent with our earlier reported results, MGE-IPs survive and migrate following transplantation in α4^+/+^ (Figure 2C) and α4^−/−^ (Figure 2D) mice. In α4^+/+^ and α4^−/−^ mice, the transplanted MGE-IPs readily migrated throughout the cortex as far as 1 mm from the injection site without apparent differences between the two genotypes. These migratory cells display a pattern of arborization consistent with mature neurons (Figures 2Cii,Dii).

**Figure 3:**
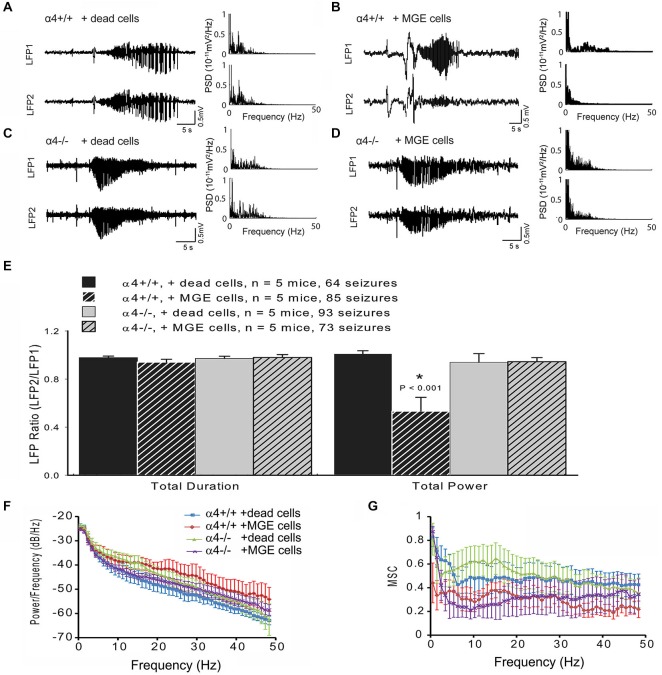
**MGE-IP transplants fail to attenuate seizure propagation in GABA_A_-R α4^−/−^ mice. (A–D)** local field potential (LFP) recording (left panel) from an α4^+/+^ mouse under control conditions **(A)** and following MGE-transplantation **(B). (C,D)** 4-AP-induced seizure-like electrical activity recorded from α4^−/−^ mice under control **(C)** and 30-days post-MGE transplant **(D)** conditions. Right panel **(A–D)** displays the power spectral density (PSD) of the LFP recording. **(E)** Summary data for ictal duration and power for each condition by genotype. **(F)** Averaged cross spectral density estimate and **(G)** averaged magnitude-squared coherence (MSC) for each condition by genotype.

As shown above (Figure 1C), deletion of the α4 subunit results in neuronal hyper-excitability. Focal neocortical ictal activity was evoked using 4-aminopyridine (4-AP) and measured (Figures 2E, 3A–D) as described (De la Cruz et al., 2011). Focal injection of 4-AP into somatosensory cortex 2 mm away from the site of MGE-IP transplant (same site as LFP1 as shown in Figure 2E) readily evoked ictal activity in α4^+/+^ (Figure 3A) and α4^−/−^ (Figure 3C) mice under control (dead cell) conditions. Ictal activity occurred simultaneously at LFP1 and LFP2, indicating that 4-AP-induced ictal activity resulted from electrical propagation by cortical spread rather than 4-AP diffusion. 4-AP-induced seizure activity was not significantly different between genotypes (Figure 3E). MGE-IP transplantation significantly reduced seizure power in α4^+/+^ (*F*_3,16_ = 9.368, *p* < 0.01; Figures 3B,E), but not in α4^−/−^ animals (Figures 3D,E), while sham injections had no effect (Figures 3A,C,E).

The raw LFP signal is a complex waveform which shows temporal variations in multiple domains, including amplitude, frequency, and phase. Fourier analysis isolates individual components of a compound waveform, and has been used to describe various aspects of the electrical seizure signal (Bronzino, 1984; Hilfiker and Egli, 1992; Blanke et al., 2000; Onoe and Nishigaki, 2004; Tsuchiya and Kogure, 2011). To analyze ictal activity beyond the standard measures of duration and total power, we also performed FFT analyses for cross power spectrum and coherence, which are additional means of analyzing the relationship between Fourier-transformed domains of the underlying electrical waveform (Bronzino, 1984; Davey et al., 2000). FFT analysis showed that cross spectral density estimate (*F*_3,16_ = 1.017, *p* > 0.05; Figure 3F) and MSC (*F*_3,16_ = 76.116, *p* > 0.05; Figure 3G) of paired LFP did not significantly differ between the four groups, indicating that absence of the α4 subunit did not fundamentally change seizure domain frequency. These data indicate that the I_tonic_ generated by GABA_A_-R α4 subunit expression is required for the ictal power reduction following MGE-IP transplantation.

## Discussion

Synaptic receptors mediate phasic inhibition whereas extrasynaptic receptors mediate tonic inhibition (Stell and Mody, 2002; Mody and Pearce, 2004; Farrant and Nusser, 2005; Belelli et al., 2009). Identification of the GABA_A_-R α4 subunit as sufficient for the reduction in ictal activity following MGE-IP transplantation suggests that extrasynaptic α4 subunit-containing GABA_A_-Rs are important targets for treating seizures arising in cortical, and perhaps subcortical, structures (Cope et al., 2009). MGE-IP transplants were shown to increase both phasic and tonic currents in cortical pyramidal neurons (Baraban et al., 2009). Augmentation of tonic currents was reported to be highly-efficient in enhancing inhibition (Bai et al., 2001). Our data emphasize the importance of extrasynaptic GABA_A_-Rs which contain the α4 subunit in constraining cellular and system level excitability. Our findings are also consistent with the observation that mutations associated with both febrile seizures and childhood absence epilepsy compromise surface expression of GABA_A_-Rs and reduce tonic GABA currents but do not affect GABA-mediated synaptic events (Eugène et al., 2007).

Is it possible that other GABA_A_-R configurations might contribute to the observed effect? δ subunit-containing GABA_A_-Rs are exclusively extrasynaptic (Nusser et al., 1998; Wei et al., 2003; Sun et al., 2004), and mediate tonic inhibition in dentate gyrus granule cells (Nusser and Mody, 2002; Stell et al., 2003; Wei et al., 2004; Maguire et al., 2005), cerebellar granule cells (Watts and Thomson, 2005), thalamic neurons (Belelli et al., 2005; Cope et al., 2005; Jia et al., 2005), and cortical pyramidal neurons (Scimemi et al., 2006). α4 and δ subunits show overlapping patterns of distribution (Peng et al., 2002), and the δ subunit normally partners with either α4 or α6 subunits (Jones et al., 1997; Sur et al., 1999; Jia et al., 2005), and while α1-δ subunits co-assemble in heterologous expression systems (Feng and Macdonald, 2004; Feng et al., 2004; Kaur et al., 2009; Baur et al., 2010), the data for such expression in neurons, while extant, is less extensive (Glykys et al., 2007).

Although the α1 subunit is well expressed in cortical neurons (Fritschy and Möhler, 1995; Araujo et al., 1996; Dunning et al., 1999; Pirker et al., 2000; Hörtnagl et al., 2013), it does not appear to contribute to the formation of α1-δ extrasynaptic GABA_A_-Rs in cortical neurons for the following reasons: (1) this current is strongly enhanced by THIP (Drasbek and Jensen, 2006; Drasbek et al., 2007; Vardya et al., 2008), and we have previously shown that α1-δ GABA_A_-Rs are relatively unaffected by the low concentrations of THIP used in those experiments (Jia et al., 2005), and more importantly; (2) there is no detectable tonic current in cortical neurons from α4^−/−^ mice (Figure 1).

Of note, there is no evidence of compensatory up-regulation in α5 or α1 subunit expression in α4^−/−^ mice, and as might be predicted, there is a significant concomitant decrease in δ subunit expression (Suryanarayanan et al., 2011); the latter effect is consistent with the decrease in δ subunit expression that is seen in α6^−/−^ mice (Jones et al., 1997), which is the other major α subunit to contribute to extrasynaptic GABA_A_-Rs. Together, those data emphasize the importance of α4 subunits in the formation of extrasynaptic GABA_A_-Rs which also contain the δ subunit. While the potential contribution of other subunits might be worth pursuing using additional pharmacologic tools, those results would not change the fundamental conclusion that the α4 subunit contributes to the efficacy of MGE transplants in constraining seizure power.

Consistent with *in vivo* data demonstrating an enhanced sensitivity to the pro-convulsant pentylentetrazole (Chandra et al., 2008), Layer 2/3 pyramidal cells from α4^−/−^ were hyperexcitable when compared to similar neurons from α4^+/+^ mice (Figure 1C). Surprisingly, R_in_ was unchanged despite the loss the tonic current, although there is precedent for such homeostatic compensation (Brickley et al., 2001). What then, could account for the change in excitability? Action potentials primarily arise in the axon initial segment (AIS; Bender and Trussell, 2012). GABA_A_-Rs have been shown to cluster at the AIS (Christie and De Blas, 2003; Muir and Kittler, 2014), even in the absence of GABAergic innervation (Christie and De Blas, 2003). Recently, it has been shown that inhibitory input to the AIS constrains both action potential formation and epileptiform activity *in vitro*, even more robustly than perisomatic inhibition (Wang et al., 2014). Thus, if extrasynaptic GABA_A_-Rs are lost at the AIS while there is a concomitant increase in an outward somatic K^+^ conductance, there will be an increase in excitability even though the overall input resistance of the cell would be unchanged. Future additional experiments will be required to test this hypothesis.

There are limitations to the present study. The implicit assumption is that efficacy of MGE transplantation in reducing ictal activity arises from an increase in GABAergic inhibition; such inhibition can be phasic, tonic (or both). Indeed, MGE transplantation has been shown to increase both phasic and tonic inhibition onto host cortical pyramidal cells, but not host interneurons, in Kv1.1 null mice (Baraban et al., 2009). Whether such an increase occurs following MGE transplantation in our animal model remains to be determined. Similarly, it is possible than the enhancement of phasic currents might also be sufficient for the observed efficacy. The α1 subunit contributes to GABA_A_-Rs at cortical pyramidal neuron synapses in adult animals (Bosman et al., 2005), and as α1 gene deletion mice have been generated (Sur et al., 2001; Vicini et al., 2001) this hypothesis could be tested; however, phasic activity is well preserved in multiple brain regions, including the cortex, despite the loss of the α1 subunit (Vicini et al., 2001; Goldstein et al., 2002; Bosman et al., 2005). This suggests that there is a strong compensatory mechanism to preserve synaptically driven GABAergic inhibition, and so multiple mouse models would appear to be required to adequately test the role of MGE-mediated enhanced phasic inhibition. Finally, differences in cell survival and/or patterns of arborization might also account for the observed lack of transplant efficacy in α4^−/−^ mice, but the degree to which survival and arborization appear to be comparable (Figures 2C,D) suggests that this is not the case. Our results are consistent with the recently reported observation that expression of the GABA_B1_ receptor subunit is not required for the migration and laminar distribution of MGE-IPs in the neocortex following transplantation (Sebe et al., 2014).

Interestingly, merely increasing overall GABA concentration, and activating *all* GABA_A_-Rs as with vigabatrin or tiagabine, to treat acquired seizures is not sufficient given the relatively poor side-effect profile associated with this approach. Our data confirm that activation of *extrasynaptic, rather than synaptic*, GABA_A_-Rs is the basis for the reduction in seizure activity following MGE transplants, and should allow us to refine the overall approach and facilitate the translation of a promising experimental observation into a clinically valuable therapy that can be used to treat patients. Cortical interneurons, including those originating in the MGE, are heterogeneous, and are classified by their morphology, neurochemical content, and electrophysiological properties (Sultan et al., 2013). Selection of an interneuron population that preferentially enhances extrasynaptic GABA_A_-R activity may improve the efficacy of cell-based therapies for the treatment of seizures arising in the cortex. Neurogliaform-type cells provide inhibitory input to cortical neurons (Tamás et al., 2003; Miyoshi et al., 2010) in a non-synaptic, spatially non-specific manner that can activate extrasynaptic GABA_A_-Rs (Oláh et al., 2009). Whether transplantation of neurogliaform interneurons can reduce seizure activity more effectively than a mixed population of interneurons remains to be tested once methods to obtain a pure population of neurogliaform progenitors is developed. Lastly, our data reveal a potentially novel target for drug development, which could be exploited as an alternative and/or complimentary approach to cell-based therapies.

## Author Contributions

MKJ: experimental design, data collection/analysis, manuscript preparation/editing, approved final approval. SK: experimental design, data collection/analysis, manuscript preparation/editing, approved final manuscript. MZ: experimental design, data collection/analysis, manuscript preparation/editing, approved final manuscript. MI: experimental design, data collection/analysis, manuscript preparation/editing, approved final manuscript. THS: experimental design, manuscript editing, approved final manuscript. SAA: experimental design, manuscript editing, approved final manuscript. GEH: experimental design, manuscript editing, approved final manuscript. PAG: experimental design, manuscript preparation/editing, approved final manuscript. Collectively the Authors agree to be accountable for all aspects of the work in ensuring that questions related to the accuracy or integrity of any part of the work are appropriately investigated and resolved.

## Conflict of Interest Statement

The authors declare that the research was conducted in the absence of any commercial or financial relationships that could be construed as a potential conflict of interest. We confirm that the all authors adhere to the International Committee of Medical Journal Editor’s position on issues involved in ethical publication and affirm that this report is consistent with those guidelines.

## Footnotes

^1^http://www.nc3rs.org.uk/
